# Automation of a flow injection system for the determination of dissolved silver at picomolar concentrations in seawater with inductively coupled plasma mass spectrometry

**DOI:** 10.1155/S1463924603000166

**Published:** 2003

**Authors:** Jose L. Barriada, Jason B. Truscott, Eric P. Achterberg

**Affiliations:** School of Earth Ocean and Environmental Sciences University of Plymouth Plymouth PL4 8AA UK

## Abstract

An automated flow injection system for the determination of dissolved silver at ultratrace concentrations in seawater, and controlled under LabVIEW™, is described. The flow injection system allows online processing of seawater samples before their analysis using a magnetic sector inductively coupled plasma mass spectrometry (MS-ICP-MS) instrument. Samples were analysed with a minimum amount of manipulation, thereby reducing the risk of contamination. In addition, the flow injection approach with incorporation of an anion exchange minicolumn allowed ready removal of analytical interferences caused by the saline matrix. The software allowed full control of all flow injection components (valves and pumps) and removed manual time control and, therefore, operator errors. The optimized system was capable of five sample injections per h, including preconcentration and wash steps. The limit of detection was 0.5 pM for a 240-s sample load time, which allowed the determination of dissolved silver in open ocean waters, where picomolar concentration levels are typically encountered.


Journal of Automated Methods & Management in Chemistry
Vol. 25, No. 4 (July–August 2003) pp. 93–100

Taylor & Francis

Taylor & Francis Group

### Automation of a flow injection system for the determination of dissolved silver at picomolar concentrations in seawater with inductively coupled plasma mass spectrometry

**Jose L. Barriada, Jason B. Truscott and Eric P. Achterberg***

*School of Earth, Ocean and Environmental Sciences, University of Plymouth, Plymouth PL4 8AA, UK*

*An automated flow injection system for the determination of dissolved silver at ultratrace concentrations in seawater, and controlled under LabVIEW$^{TM}$, is described. The flow injection system allows online processing of seawater samples before their analysis using a magnetic sector inductively coupled plasma mass spectrometry (MS-ICP-MS) instrument. Samples were analysed with a minimum amount of manipulation, thereby reducing the risk of contamination. In addition, the flow injection approach with incorporation of an anion exchange minicolumn allowed ready removal of analytical interferences caused by the saline matrix. The software allowed full control of all flow injection components (valves and pumps) and removed manual time control and, therefore, operator errors. The optimized system was capable of five sample injections per h, including preconcentration and wash steps. The limit of detection was 0.5 pM for a 240-s sample load time, which allowed the determination of dissolved silver in open ocean waters, where picomolar concentration levels are typically encountered.*

### Introduction

Silver is one of the most toxic elements, surpassed only by mercury [1]. Monitoring of dissolved silver concentrations in estuarine and coastal waters is therefore of great importance for water quality management. Anthropogenic sources of silver to these waters include sewage discharges, run off from operating and disused mines, and release from contaminated sediments. Dissolved silver is taken up by marine organisms, and in oceanic environments it shows a recycled behaviour, parallel to silicate [2–4]. The determination of dissolved silver in marine waters is not without its challenges, with typical concentrations at picomolar levels and interference problems from the saline matrix affecting most analytical techniques. Consequently, a limited amount of data on dissolved silver in marine waters is available.

Several analytical techniques have been reported for the determination of dissolved silver in natural waters, including graphite furnace atomic absorption spectroscopy (GFAAS) after solvent extraction [2, 5–9], and inductively coupled plasma mass spectrometry (ICP-MS) after solvent or solid extraction [3, 10, 11]. Although voltammetric techniques have been reported for dissolved silver measurements in natural waters (including seawater) [12], they have been scarcely applied to environmental studies [13] because of the challenges posed by the low concentrations of silver in these waters.

In the present studies, flow injection (FI) coupled to MS-ICP-MS detection was the chosen technique for dissolved silver analysis. The FI approach allows automation, a high sample throughput and facilitates trace metal clean operating procedures, and the detection system exhibits a high sensitivity and minimal interference problems.

### Experimental

### *Reagents and samples*

Chemicals were obtained from VWR International Ltd (Lutterworth, UK), unless otherwise stated. Labware was cleaned following trace metal analysis procedures [14] by soaking in successive baths of 2% v/v micro-detergent (Decon) for 24 h, 6 M HCl for 1 week and 3 M $HNO_3$ (both ARISTAR) for 1 week, with thorough rinses using MilliQ (MilliPore, Molsheim, France) water between each step. All sample handling was carried out in a class 100 laminar flow hood. High purity (Sb-) $HNO_3$ was produced by sub-boiling distillation from $HNO_3$ (ARISTAR).

Silver preconcentration and removal of the saline matrix was undertaken in the FI manifold using a minicolumn (1 cm long and 85 µl internal volume; Global FIA, Inc., Fox Island, WA, USA) filled with a slurry of a strong anionic exchange resin (Dowex 1X8, 200–400 mesh; Supelco, Bellefonte, CA, USA) [11]. Non-metal frits (35 µm pore size) were fitted onto the ends of the mini-column, which was then placed in a minicolumn holder (Global FIA). The resin exchanges anions with a solution as follows:

$$R_z \overset{\oplus}{N} R_3\mathrm{Cl}^- + X^- \leftrightarrows R_z \overset{\oplus}{N} R_3 X^- + \mathrm{Cl}^-,$$

where $R_z$ is the polymer matrix of the resin and $X^-$ is an anion in the solution. In seawater, dissolved silver is present as chlorocomplexes ($AgCl_2^-$ and $AgCl_3^{2-}$) [11, 15]. During the preconcentration step, the silver complexes

* To whom correspondence should be addressed.
e-mail: eachterberg@plymouth.ac.uk http://www.tandf.co.uk/journals
DOI: 10.1080/1463924031000163724

are retained and preconcentrated by the resin in the minicolumn. In the analytical procedure, salts are subsequently washed off the minicolumn during the rinse step with MilliQ water and, finally, silver chlorocomplexes are eluted into the MS-ICP-MS using 1.2 M $HNO_3$.

Polytetrafluoroethylene (PTFE) tubing with a 0.75 mm internal diameter (Altec Products, Bude, UK) was used throughout the FI manifold for sample transport. Tygon® peristaltic pump tubing (Elkay, Basingstoke, UK) was used for $HNO_3$, and sample and MilliQ water propulsion (1.14 and 1.54 mm internal diameter, respectively).

A silver standard ($10^{-8}$ M) was prepared daily from concentrated silver solution (BD-ICP-MS 1000 ppm standard) and acidified to pH ≈ 2 using Sb-$HNO_3$. Seawater was collected in trace metal clean low-density polyethylene (LDPE) bottles (Nalgene™, Nalge Nunc International, Rochester, USA) and filtered using 0.4 µm pore size polycarbonate filters (Cyclopore™, Whatman International Ltd, Maidstone, UK). Samples were acidified to pH ≈ 2 using Sb-$HNO_3$ and the bottles were double-bagged for storage.

*Flow injection instrument*

An automated FI system (PrepLab, Fisons Instruments, Elemental Analysis, Winsford, UK) coupled with an MS-ICP-MS instrument (VG Elemental, Winsford, UK) was used for the determination of dissolved silver in seawater samples. A LabVIEW™ (National Instruments, Austin, TX, USA) graphical programming environment [16] was used to design a virtual instrument (VI) with a flexible user interface for the FI instrument control. Figure 1 shows a scheme for the coupled PrepLab FI-MS-ICP-MS system. The heart of the system is a dual six-way switching valve, which is pneumatically switched between two different positions by means of high-pressure (> 20 psi) argon gas. All ports and fittings in the dual six-way valve are made of polyether ether ketone (PEEK), an inert material suitable for ultratrace metal analysis. The FI system analysis steps are described in table 1; a full cycle consists of four main steps: Step 1: a prerinsing step of 30 s during which MilliQ water is passed through the minicolumn and diluted $HNO_3$ (≈ 0.12 M) is aspirated into the MS-ICP-MS; Step 2: a loading step of 240 s during which a seawater sample is passed through the minicolumn to retain silver complexes on the resin (during this step diluted $HNO_3$ is aspirated into the MS-ICP-MS); Step 3: a rinsing step of 120 s during which MilliQ water is passed through the minicolumn to wash out salts (again diluted $HNO_3$ is aspirated into the MS-ICP-MS); and Step 4: an elution step of 220 s during which $HNO_3$ (≈ 1.2 M) is passed through the minicolumn and silver chlorocomplexes are eluted into the MS-ICP-MS. During the last 10 s of Step 4, valve 2 is switched to preload a new sample for the next run. Throughout the analysis, the pump speeds were adjusted to a flow rate of 3 ml min$^{-1}$ for sample transport and 2 ml min$^{-1}$ for acid elution.

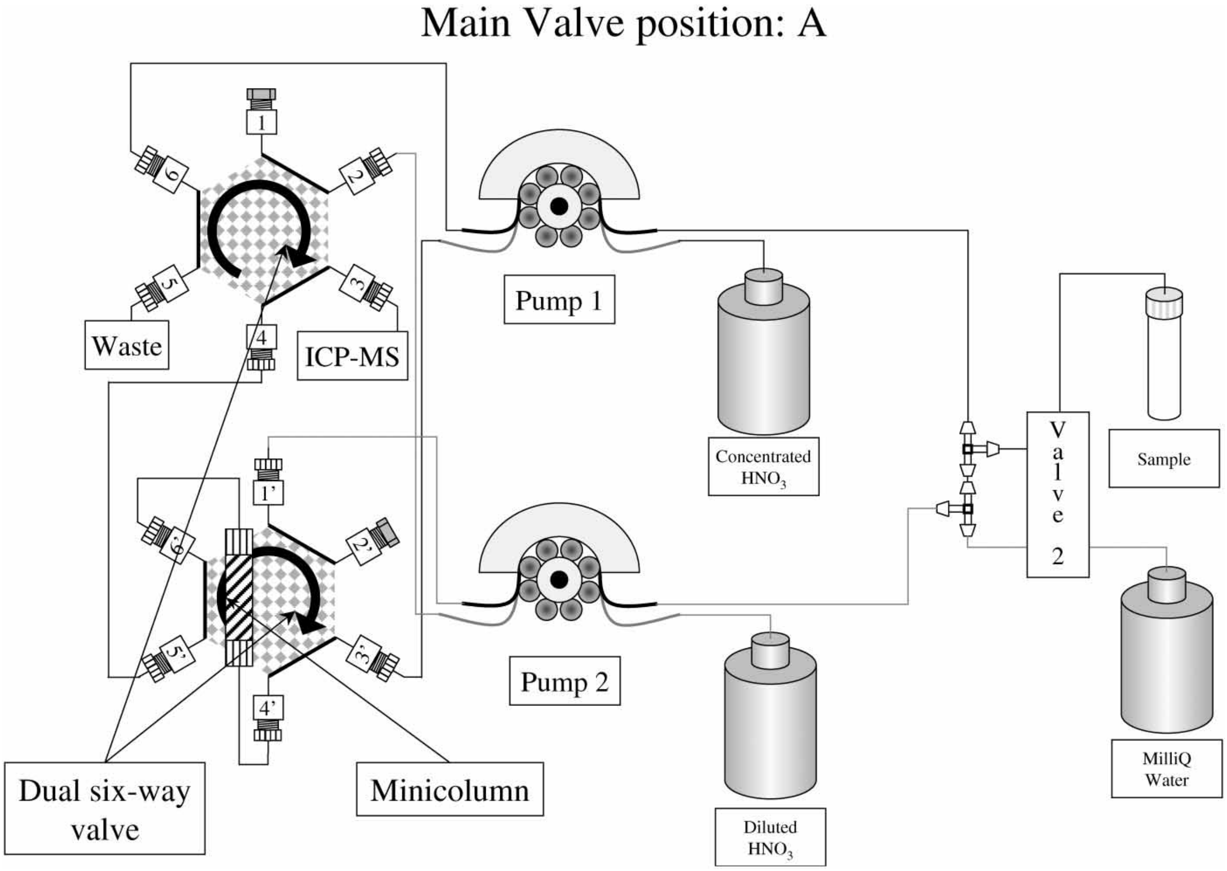

*Figure 1. System layout for FI coupled to the MS-ICP-MS instrument for the determination of dissolved silver in seawater.*

*Table 1. Time sequence for a single analysis cycle.*

| Time in operation (s) | Pumps 1 | Pumps 2 | Valve 2 position | Dual six-way valve position | Step |
|---|---|---|---|---|---|
| 30 | off | on | off | B | prerinse |
| 240 | off | on | on | B | load |
| 120 | off | on | off | B | wash |
| 220 | on | off | off[a] | A | elution |
| Back to Step 1 | | | | | |

[a]Switch valve 2 is changed to 'on' during the last 10 s, and a new sample is preloaded onto column..
For the description of valves and pumps, see figure 1.

*Table 2. MS-ICP-MS settings.*

| | VG axiom MS-ICP-MS |
|---|---|
| ICP | |
| Forward power (W) | 1225 |
| Plasma gas ($l\,min^{-1}$) | 13.5 |
| Auxiliary gas ($l\,min^{-1}$) | 0.85 |
| Nebulizer gas ($l\,min^{-1}$) | 0.77 |
| Sample flow ($ml\,min^{-1}$) | 2 |
| Torch | Fassel (quartz) |
| Nebulizer | concentric (quartz) |
| Spray chambers | cyclonic + bead impact |
| Interface | |
| Sampler | Ni |
| Skimmer | Ni |
| Mass spectrometer | |
| Ion masses ($m/z$) | $^{107}Ag$ |
| Resolution | 400 |
| Data acquisition | |
| Single ion monitoring | 110 points |
| Dwell time (ms) | 2000 |

The MS-ICP-MS instrument was operated in a low resolution mode and tuned (gas flows and torch position) using a 1 ppb solution of $^{115}In$ before experiments to obtain the optimal instrument sensitivity. Table 2 presents typical argon gas flows and MS-ICP-MS settings obtained following tuning of the instrument. MS-ICP-MS measurements were undertaken using a single ion monitoring mode for $^{107}Ag$ with a dwelling time of 2000 ms and a registration of 110 points. Before and during analysis, MilliQ water aliquots were run to check the background MS-ICP-MS signal and to rinse the minicolumn and FI tubing thoroughly.

### *Flow injection instrument–computer interface*

FI system control was achieved using a RS232 16-line I/O card—SIO (Intec Associates Ltd, Woking, UK) (figure 2) housed in an interface box (Ruthern Instruments Ltd, Bodmin, UK), which was connected to the serial port of a laptop computer via a serial port laplink cable (DB9/DB25-DB9/DB25). The connection between the interface box and the FI system was made using a 25-pin cable. The interface box obtained power from the PrepLab FI system. VI software was developed in-house and written in LabVIEW™ (Version 6, National Instruments Corp., Austin, TX, USA). The LabVIEW™ VI front panel (figure 3) contains ready-to-use switches, buttons and controls to operate valves and pumps, and allows the definition of sample treatment times. The status of the sample process is displayed on the right-hand side of the front panel (figure 3). The VI wiring diagrams are shown in figures 4 and 5, with figure 4 showing the initialization, analysis step definition and closure procedures, and figure 5 the main LabVIEW™ loop and sequences of the VI.

In the initialization process, the VI takes control of the pumps (stopped), the dual six-way valve (switched to position A), and valves 1 and 2 (switched off). The FI system is set to the standby mode and either a manual (e.g. switching pumps and/or valves) or a programmed step for sample analysis cycle can be defined through the front panel. The sample analysis cycle is started using the 'Finish steps?' button (figure 3, bottom left-hand side).

The analysis cycle is halted at the elution step, and after this alert is acknowledged, the cycle continues, the MS-ICP-MS single-ion monitor register is started, and concurrently the preconcentration of a new solution is

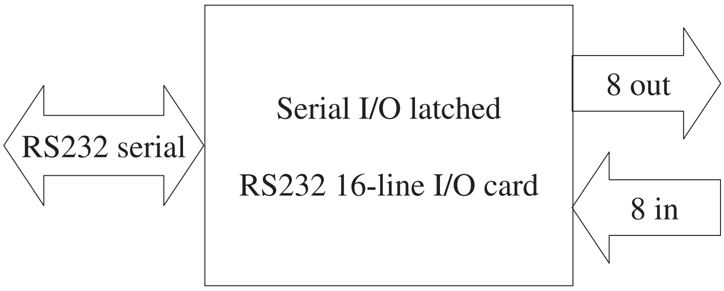

*Figure 2. FI system interface.*

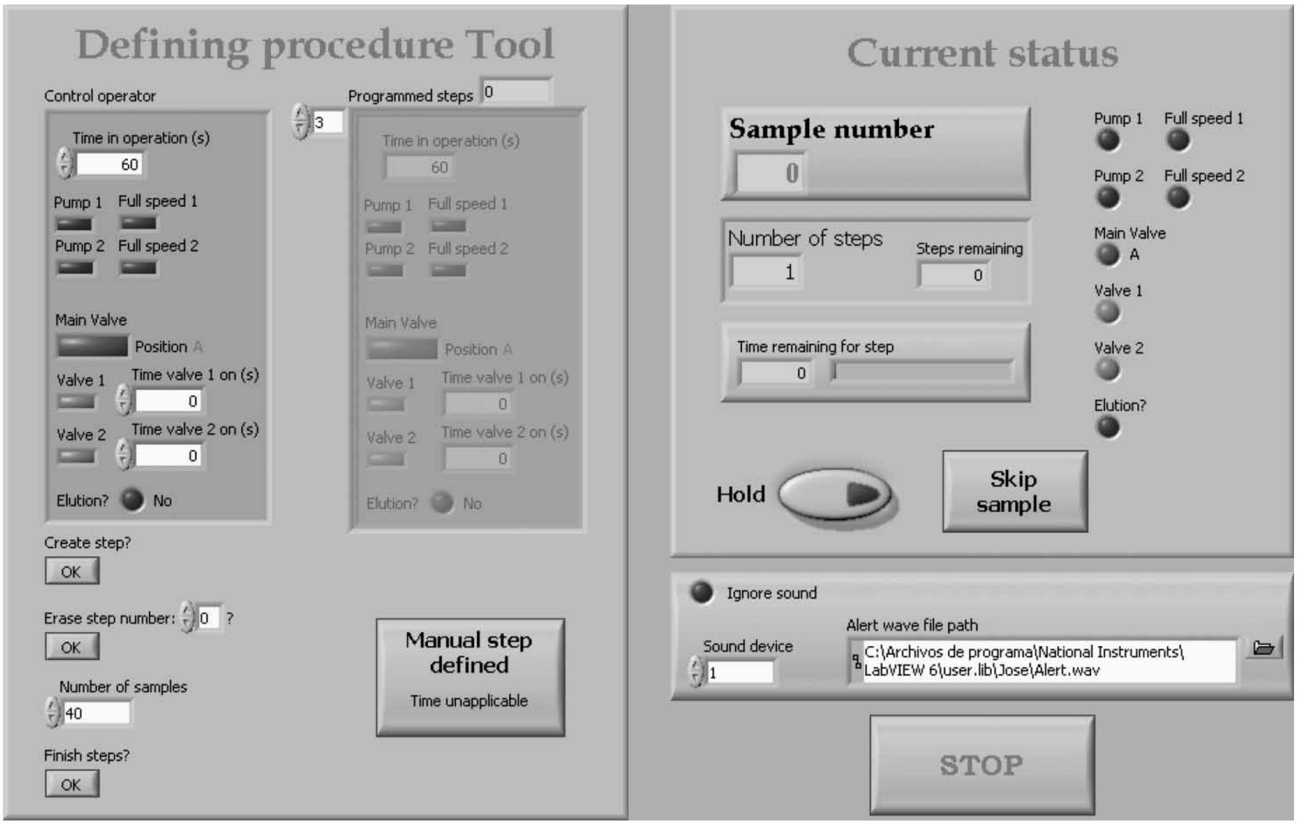

*Figure 3. LabVIEW$^{TM}$ graphical user front panel for the automated virtual instrument.*

commenced. Figure 5 shows the LabVIEW VI time-controlled sequence structure in which the valves and pumps are set either to the true or false stage to switch them on or off. Although the main valve port connections restrict the use of pumps in the employed setup (e.g. pump 2 cannot be used with main valve in position A; figure 1), the LabVIEW VI has been designed to be flexible and allows one to use all possible pump/valve combinations in the FI system.

*Environmental application*

Figure 6 shows a typical background (MilliQ water) and sample peak signal chromatogram. The peak area of the elution profiles was determined by means of a macro created for Microsoft Excel® (Microsoft Corporation, Redmond, WA, USA), using the trapezoid sum rule. Dissolved silver concentrations in seawater samples were determined from the standard addition plot by extrapolation. An example of a standard addition plot for a seawater sample is shown in figure 7, and shows excellent linearity for the silver peak area signal over a concentration range of 6–33 pM. The limit of detection for the dissolved silver analysis was calculated from the regression line as three times the standard error of the fitting [17] and amounted to 0.5 pM.

The optimized FI-MS-ICP-MS method has been applied to samples from different marine waters. Figure 8 shows a depth profile of dissolved silver in seawater samples collected at station 28 (46:00 01 N, 8:00 02 W) during the *RV Pelagia* cruise (March 2002) using Go-Flo bottles (General Oceanics, Inc., Miami, FL, USA). Dissolved silver concentrations were found to increase from below 10 pM in the top 1000 m to about 20 pM in deep waters. The dissolved silver concentrations, therefore, showed a recycled behaviour with a similar trend and concen-

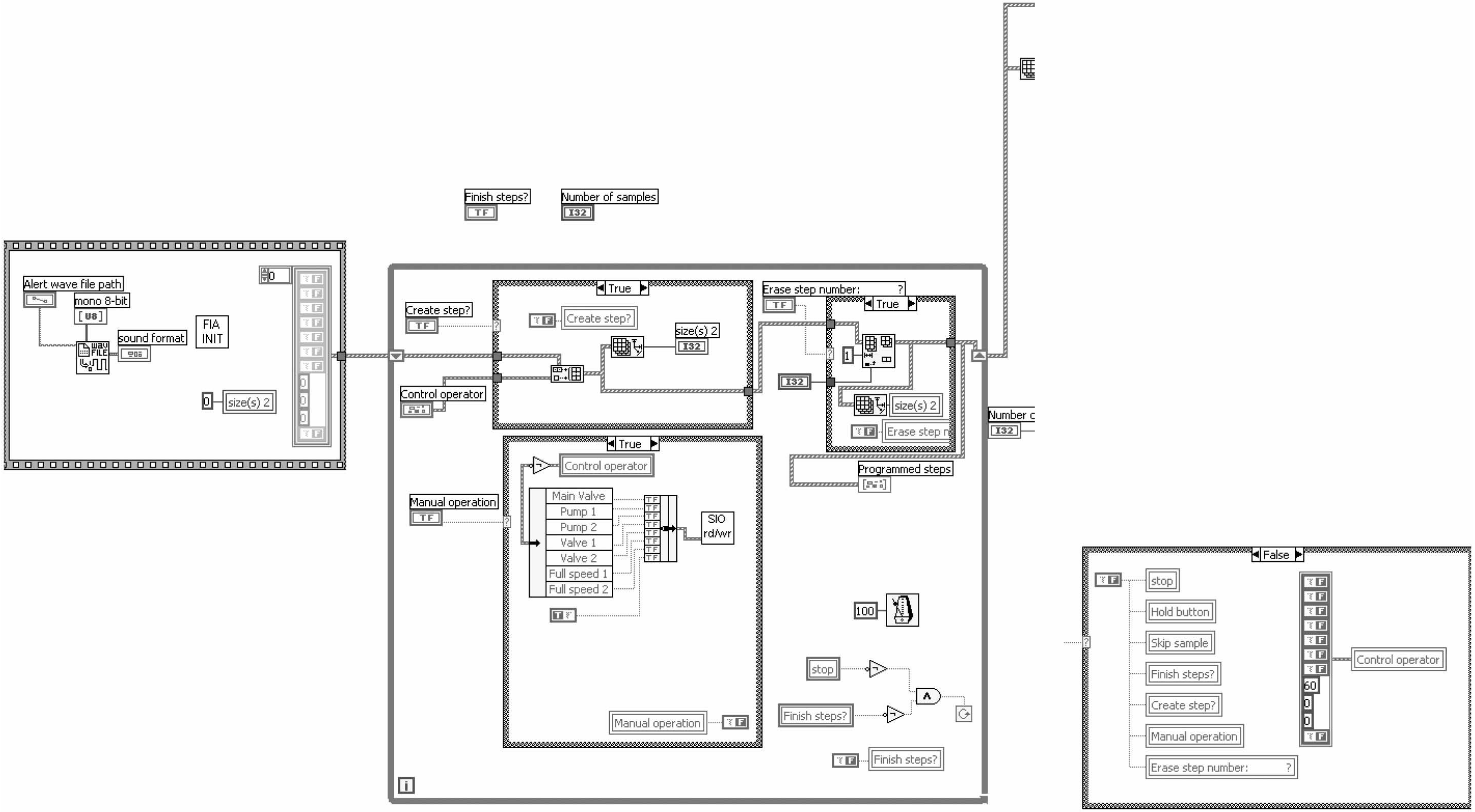

*Figure 4. Graphical code for instrument control. The left-hand side shows the initialization and the step-defining sequences; the right-hand side shows the closure sequence.*

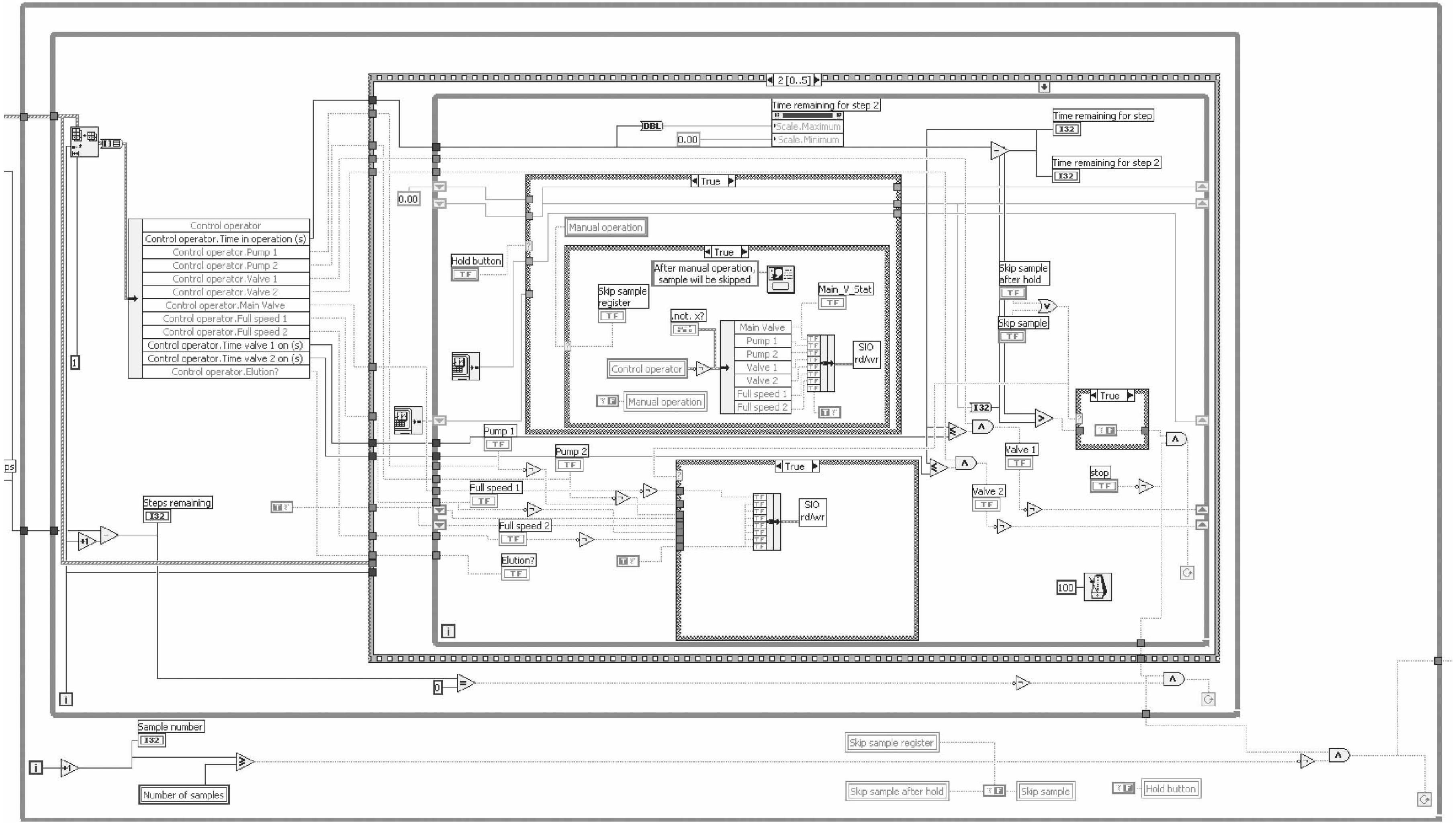

*Figure 5. Graphical code for the instrument control (continued). The main block graphical code is shown with the sequence structure operating during the sample processing. This code, together with codes shown in figure 4, drives the functions shown on the* Front Panel *in figure 3.*

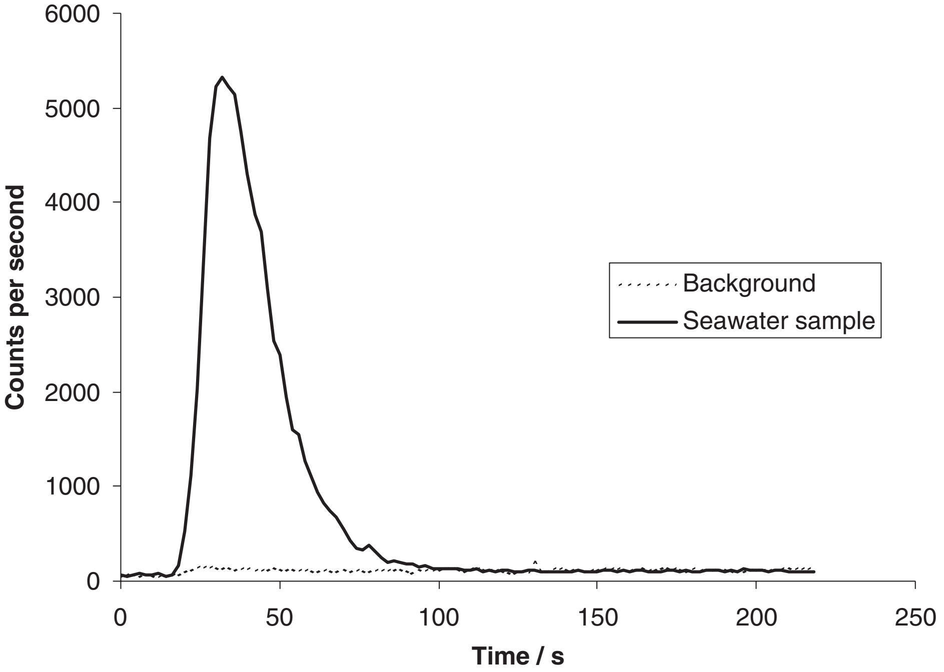

*Figure 6. Typical elution chromatogram for the background and dissolved silver signal.*

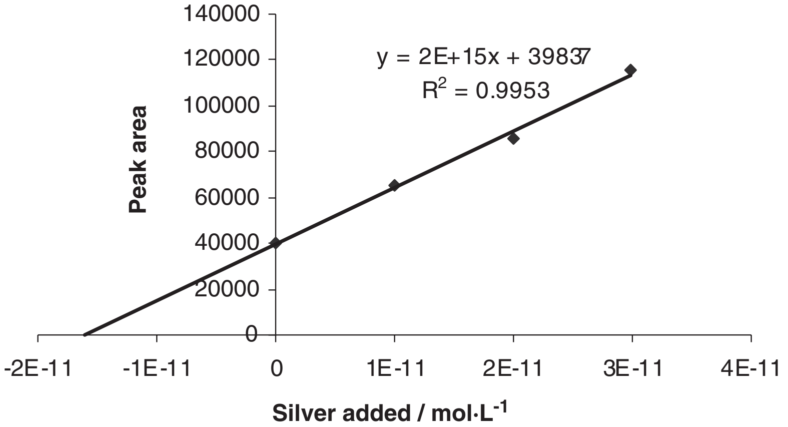

*Figure 7. Standard addition plot for dissolved silver.*

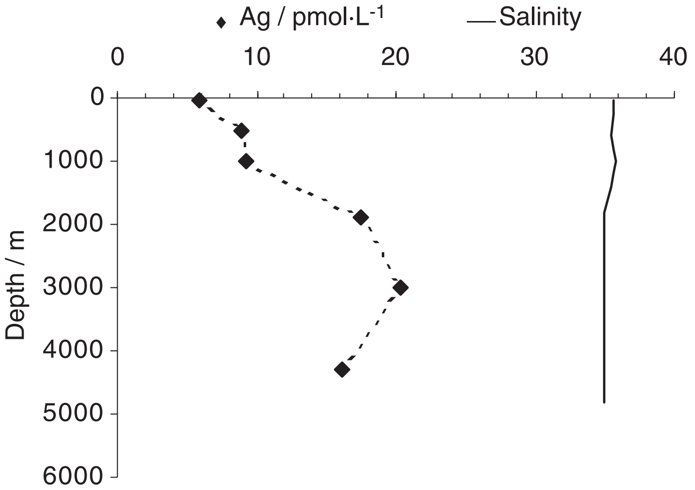

*Figure 8. Depth profiles of dissolved silver and salinity at station 28 in the north-east Atlantic Ocean.*

tration range as reported for the north-east Pacific by Martin *et al.* [18]. Dissolved silver concentrations in deep waters were higher than those reported by Flegal *et al.* [2] for the Eastern Atlantic (up to 10 pM), but still in the same order of magnitude of typical values of dissolved silver observed in open ocean waters.

### Conclusions

The automated virtual instrument developed here uses flow injection with MS-ICP-MS detection for the determination of dissolved silver in seawater. The FI-MS-ICP-MS set-up allows dissolved silver analysis in seawater with a minimum of sample manipulation, thereby reducing the risk of contamination, which poses the main problem in ultratrace metal analysis. The automated approach improves sample throughput and time control of the analytical steps. The detection limit of 0.5 pM allows the determination of silver in all marine environments, including open ocean waters. The developed methodology will allow the study of the distribution and behaviour of dissolved silver in marine ecosystems, for which a very limited amount of data are available.

### Acknowledgements

The authors thank John Wood (Ruthern Instruments Ltd) for construction of the main control interface and Simon Ussher for sample collection. Research was supported by a Marie Curie Fellowship of the European Community programme Energy, Environment and Sustainable Development under Contract Number EVK3-CT-2001-50004.

### References

1. Ratte, H. T., *Environ. Toxicol. Chem.*, **18** (1999), 89.
2. Flegal, A. R., Sanudowilhelmy, S. A. and Scelfo, G. M., *Mar. Chem.*, **49** (1995), 315.
3. Ndung'u, K., Thomas, M. A. and Flegal, A. R., *Deep-Sea Res. Part II LML Top. Stud. Oceanogr.*, **48** (2001), 2933.
4. Rivera-Duarte, I., Flegal, A. R., Sanudo-Wilhelmy, S. A. and Veron, A. J., *Deep-Sea Res. Part II LML Top. Stud. Oceanogr.*, **46** (1999), 979.
5. Smith, G. J. and Flegal, A. R., *Estuaries*, **16** (1993), 547.
6. Miller, L. A. and Bruland, K. W., *Environ. Sci. Technol.*, **29** (1995), 2616.
7. RiveraDuarte, I. and Flegal, A. R., *Mar. Chem.*, **56** (1997), 15.
8. Breuer, E., Sanudo-Wilhelmy, S. A. and Aller, R. C., *Estuaries*, **22** (1999) 603.
9. Herrin, R. T., Andren, A. W. and Armstrong, D. E., *Environ. Sci. Technol.*, **35** (2001), 1953.
10. Zhang, Y., Amakawa, H. and Nozaki, Y., *Mar. Chem.*, **75** (2001), 151.
11. Yang, L. and Sturgeon, R. E., *J. Anal. At. Spectrom.*, **17** (2002), 88.
12. Von Wandruszka, R., in Alfassi, Z. B. and Wai, C. M. (eds), *Preconcentration Techniques for Trace Elements* (Boca Raton: CRC Press, 1992), 133.
13. Shpigun, L. K. and Kopytova, N. E., *Ind. Lab.*, **63** (1997), 129.
14. Achterberg, E. P., Holland, T. W., Bowie, A. R., Fauzi, R., Mantoura, C. and Worsfold, P. J., *Anal. Chim. Acta*, **442** (2001), 1.
15. Reinfelder, J. R. and Chang, S. I., *Environ. Sci. Technol.*, **33** (1999), 1860.
16. http://www.ni.com/labview/
17. Miller, J. C. and Miller, J. N., *Statistics and Chemometrics for Analytical Chemistry*, 4th edn (Harlow: Prentice-Hall, 2000).
18. Martin, J. H., Knauer, G. A. and Gordon, R. M., *Nature*, **305** (1983), 306.

